# Major cardiovascular events under biologic psoriasis therapies: a 19-year real-world analysis of FAERS data

**DOI:** 10.3389/fimmu.2024.1349636

**Published:** 2024-02-07

**Authors:** Lingqing Ding, Congqin Chen, Yongkuan Yang, Xiaoting Zhang

**Affiliations:** ^1^ Department of Pharmacy, Xiamen Cardiovascular Hospital, Xiamen University, Xiamen, China; ^2^ School of Electrical Engineering and Automation, Xiamen University of Technology, Xiamen, China

**Keywords:** biologics, psoriasis, major adverse cardiovascular adverse events, disproportionality analysis, safety signal

## Abstract

**Objective:**

Over the years when biologic psoriasis therapies (TNF inhibitors, IL-12/23 inhibitors, IL-23 inhibitors, and IL-17 inhibitors) have been used in psoriasis patients, reports of major cardiovascular events (MACEs) have emerged. This study aims to investigate the association between MACEs and biologic psoriasis therapies by using information reported to the US Food and Drug Administration Adverse Event Reporting System (FAERS).

**Methods:**

FAERS data (January 2004 to December 2022) were reviewed. For each drug–event pair, the proportional reporting ratio (PRR) and the multi-item gamma Poisson shrinker (MGPS) algorithms were used to identify drug–adverse event associations.

**Results:**

We filtered the query for indication and identified 173,330 reports with psoriasis indication in FAERS throughout the analyzed time frame. MACEs occurred in 4,206 patients treated with biologics. All the four biological classes had an elevated and similar reporting rates for MACEs relative to other alternative psoriasis treatments (PRR from 2.10 to 4.26; EB05 from 1.15 to 2.45). The descending order of association was IL-12/23 inhibitors>IL-17 inhibitors>IL-23 inhibitors>TNF inhibitors. The signal strength for myocardial infarction (PRR, 2.86; χ^2^, 296.27; EBGM 05, 1.13) was stronger than that for stroke, cardiac fatality, and death. All the biological classes demonstrated a little higher EBGM 05 score≥1 for the MACEs in patients aged 45–64 years. The time-to-onset of MACEs was calculated with a median of 228 days.

**Conclusions:**

Analysis of adverse event reports in the FAERS reflects the potential risk of MACEs associated with the real-world use of biological therapies in comparison to other alternative psoriasis treatments. Future long-term and well-designed studies are needed to further our knowledge regarding the cardiovascular safety profile of these agents.

## Highlights

- The Federal Adverse Event Reporting System (FAERS) is a US Food and Drug Administration (FDA) spontaneous reporting database, and it collects hundreds of millions of drug adverse events reports worldwide submitted by health professionals, consumers, and manufacturers.
- Biologic regimens are a revolutionary discovery in the field of moderate-to-severe psoriasis treatment. Over the years when biologics have been applied, reports of major cardiovascular events (MACEs) have emerged.
- Analysis of adverse event reports in the FAERS reflects the potential risk of MACEs associated with the real-world use of biological therapies in comparison to other alternative psoriasis treatments.
- In clinical practice, detailed cardiovascular history and measures like electrocardiogram and cardiac ultrasonography may be necessary, when prescribing biologic agents in patients with high cardiovascular risk factors.

## 1 Introduction

Psoriasis is a chronic inflammatory multi-systemic skin disease associated with several extracutaneous complications (1). In general, this disease is alleviated by four therapies (phototherapy, topical therapy, traditional systemic therapy, and biological therapy). Topical therapy includes the use of corticosteroids, vitamin D3 analogs, and calcineurin inhibitors. Traditional systemic therapy, including methotrexate and ciclosporin, serves as as first-line agents for moderate to severe psoriasis. Biological therapy (including tumor necrosis factor [TNF] inhibitors, interleukin [IL]-12/23 inhibitors, IL-23 inhibitors, and IL-17 inhibitors) has been performed with high efficacy and safety as compared to the other three therapies. They can substantially suppress inflammatory cytokines activity and thus represent a revolutionary discovery in the treatment of moderate to severe psoriasis treatment (2–4).

While the benefit of biologics is well established for the remission of psoriasis, reports of major cardiovascular events (MACEs) have emerged over the years when biologics have been applied (5–7). In this regard, significant concern has been raised about MACEs secondary to biologic psoriasis therapies. However, the evidence remains mixed regarding the impacts of biological therapies on cardiac outcomes (8–15). Adverse events after use of biological agents most frequently reported through clinical trials were infections, nasopharyngitis, and headache (16, 17). A comprehensive examination and meta-analysis involving 38 randomized controlled trials (9) concluded that biologics (referring to TNF inhibitors, IL-17 inhibitors, and IL-12/23 inhibitors) did not increase the risk of MACE over the short term. Nonetheless, contradictory results emerge from real-world investigations into the safety of biological agents. In 2011, the clinical trials for the IL-12/23 inhibitor, briakinumab, was discontinued before marketing after the submitted reports of serious cardiovascular adverse events associated with its use (e.g., myocardial infarction, cardiac death) (18, 19). Recently, after analyzing real-world post-marketing data sourced from the FDA Adverse Event Reporting System, Woods and collaborators identified a potential signal for cerebrovascular accidents linked to risankizumab (20). Similarly, a descriptive analysis based on safety reports from individual cases within the European Economic Area and the United Kingdom in EudraVigilance revealed that cardiac disorders occupied 10.9% of serious adverse events suspected of being caused by risankizumab during the past 3 years (21).

Moreover, as these biologics vary regarding their characteristics such as action and safety (22, 23), are there any differences between the different biological classes with regard to safety signals on MACEs? It is hard to answer, as yet, evidence on their comparative cardiovascular safety remains conflicting based on previous clinical trials and biomarkers studies (2, 14, 24, 25). We know that conducting long-term prospective placebo-controlled trials in patients with psoriasis is either unavailable or considered ethically impractical because they require regular anti-inflammatory therapy. Thus, in this study, a retrospective disproportionality analysis based on the FDA Adverse Event Reporting System (FAERS), one of the most extensive publicly accessible spontaneous reporting systems, was executed to evaluate the possible association between biologic psoriasis therapies and MACEs.

## 2 Method

### 2.1 Data source

FAERS collects hundreds of millions of drug adverse events reports worldwide submitted by health professionals, consumers, and manufacturers. We chose the FAERS dataset as the data source for this study, and the analytical period was from January 2004 to December 2022. To enhance the reliability and validity of our results, only records submitted by professional health workers (e.g., physicians, pharmacists, and other health professionals) were included. To avoid potential confounders in causality assessments such as indication, the analysis was refined by retaining only reports with an expanded psoriasis indication. The raw FAERS data were cleaned, mapped, de-duplicated, and normalized by SQL server Data Mining.

### 2.2 Definition of suspected drugs and control groups

The licensed biologics for psoriasis, including TNF-α inhibitors (Adalimumab, Etanercept, Infliximab, Certolizumab, and Golimumab), IL-23 inhibitors (Guselkumab, Risankizumab, and Tildrakizumab), IL-12/23 inhibitors (Ustekinumab), and IL-17 inhibitors (Ixekizumab, Secukinumab, Brodalumab) were selected as the suspected drugs in this study. The names are listed in **Supplementary Table S1**.

Inclusion of reports was limited to only those cases where a biologic drug was identified as “the primary suspect” or”secondary suspect”. The control groups were comprised of the population who were indicated with psoriasis and receiving other psoriasis systemic therapies except biological agents.

### 2.3 Definition of adverse events

There is no standard definition for MACEs, and the definition varies by study. In our study, MACEs was a composite endpoint including nonfatal myocardial infarction, nonfatal stroke, and cardiovascular death (26, 27). In order to identify a representative MACEs, the Medical Dictionary for Regulatory Activities (MedDRA, version 25.0) and Standardized MedDRA Queries (SMQs) were routinely adhered to. Subsequently, four SMQs were chosen, namely, myocardial infarction, stroke, cardiac fatality, and death, which are specified in **Supplementary Table S2**. In addition, the terminology for adverse events was established using the preferred terms (PTs) within the MedDRA registration.

### 2.4 Signal detection

A series of analyses were conducted to detect a disproportionate reporting with the use of biological therapies for MACEs. In the attempt to avoid the potential impact of the basic psoriasis disease process on the outcome of cardiovascular events and ensure balance in baseline characteristics among the study population, the following analyses used the reports limited to non-biologics for a similar indication (psoriasis) as the comparator group (20, 28, 29). First, comparator-restricted analyses were performed through which the MACEs reporting with the four biological classes was in comparison to MACEs reporting with other active comparator anti-psoriasis drugs. Then, two subgroup analyses were conducted utilizing the stratified Bayesian analysis (Multi-Item Gamma Poisson Shrinker, MGPS) to reduce the influence of confounding variables (such as age and sex) on cardiovascular diseases and to evaluate the ADR profile in different age/sex groups for all the selected drug–event pairs. The MGPS analysis was conducted separately for four cohorts of patients based on age cutoffs: 0–17, 18–44, 45–64, and ≥65 years. Meantime, the MGPS analysis also stratifies the data into three sex categories (male, female, and unknown) to evaluate potential sex-related variations in adverse event profiles.

This study is a case–non-case study. Reports of the adverse reaction of concern are referred to “cases”, while other reports are designated as “non-cases” (30). Disproportionality analysis (proportional reporting ratio, PRR) and MGPS were calculated to identify drug–event combinations with reporting rates higher than expected versus other drugs among patients with psoriasis indication. First, a common metric of association, the PRR, is used. It is a commonly used disproportionality method and calculated using the formula PRR=a/(a+b)/[c/(c+d)], where a corresponds to the number of reports of MACEs for biologics, b represents the number of reports for biologics without reporting MACEs, c is the number of the reports of MACEs for all other drugs, and d signifies the count of reports for all other drugs that do not report MACEs. The PRR has the advantage of being self-explanatory, straightforward to compute, and easy to understand. It has the disadvantage of being highly unstable when the reports for a particular drug–event combination are few (that counts a, b, and c are very small). To overcome this drawback, a more robust method such as MGPS is used that is typically worked with the PRR in large safety database. The MGPS uses an empiric Bayesian model that provides an adjusted value of an observed:expected ratio referred to as the empiric Bayes geometric mean (EBGM), which can efficiently discover potential drug–event associations in FAERS.

For PRR, a signal is identified when PRR≥2, χ2≥4, and the number of reports≥3 (31). The lower and upper 90% confidence interval for the EBGM is defined by EB05 and EB95. EB05≥2 is a common signal definition that means a drug–event in question is at least twice the expected ratio relative to the comparator drugs–events in the database. However, for serious events such as myocardial infarction and stroke, the criterion of EB05≥2 to define a signal would be too lax. For serious events, more rigorous signaling requirements would be appropriate to detect associations that exceed the expected values for such serious events (32, 33), which is also recommended in a guidance document from FDA (34). Thus, in our study, to account for the severity of MACEs, we selected EB05>1 as the signal criterion. The MACEs signals could be determined when both the PRR and EBGM results yielded significance. When both PRRs and EBGM values were >1, however, if the criteria were not met, this was interpreted as suggestive of a non-significant trend toward higher-than-expected reporting rates. To identify reporting differences among drugs, we use confidence intervals (EB05 and EB95) for comparisons, provided that they do not overlap.

## 3 Results

### 3.1 Data overview

During the study period, a total of 20,433,868 AE reports were collected initially. Then, we filtered the query for an expanded psoriasis indication and identified 173,330 reports in FAERS during the study period. Overall, 167,417 AEs were found to be associated with biologics for psoriasis. Among them, MACEs occurred in 4,206 reports where the biologics were identified as the suspected drugs.

### 3.2 General characteristics

Men accounted for the majority of the cases (2,565 reports, 60.98%), while women accounted for 1,478 (35.14%) reports. Of the reports where age was documented, the median age was 60 (interquartile range, IQR, 52–67) years. The largest proportion of reports (1,810 reports, 43.03%) came from individuals aged 45–64 years. The country with the highest number of reported cases was the United States (1,680 reports, 39.94%). MACEs outcomes often resulted in hospitalization (47.91%) or death (17.48%). The characteristics of the MACEs reports for biological agents are presented in **Table 1**.

**Table 1:** Characteristics of biologics-associated MACEs reports submitted by health professionals in the FAERS database (2004 Q1–2022 Q4).

Reports n (%)					
	Total	TNF inhibitor	IL-17 inhibitor	IL-23 inhibitor	IL-12/23 inhibitor
Cases number	4206	2518	851	194	808
Sex					
Male	2,565 (60.98%)	1,534 (60.92%)	480 (56.40%)	124 (63.92%)	538 (66.58%)
Female	1,478 (35.14%)	891 (35.39%)	329 (38.66%)	56 (28.87%)	232 (28.71%)
Missing	163 (3.88%)	93 (3.69%)	42 (4.94%)	14 (7.22%)	38 (4.70%)
Age (years)					
Median(IQR)	60 (52,67)	60 (52,67)	60 (52,67)	62 (52,69)	58 (50.66)
0–17 years	3 (0.07%)	1 (0.04%)	0 (0.00%)	0 (0.00%)	2 (0.25%)
18–44 years	377 (8.96%)	238 (9.45%)	54 (6.35%)	14 (7.22%)	82 (10.15%)
45–64 years	1,810 (43.03%)	1,076 (42.73%)	351 (41.25%)	76 (39.18%)	379 (46.91%)
≥65years	1,079 (25.65%)	674 (16.02%)	200 (4.76%)	55 (1.31%)	190 (4.52%)
Not specified	937 (22.28%)	529 (21.01%)	246 (28.91%)	49 (25.26%)	155 (19.18%)
Outcome§					
Death	690 (17.48%)	462 (19.21%)	114 (15.06%)	20 (11.76%)	127 (16.37%)
Life-threatening	361 (9.15%)	156 (6.49%)	103 (13.61%)	18 (10.59%)	110 (14.18%)
Hospitalization	1,891 (47.91%)	1,182 (49.15%)	332 (43.86%)	88 (51.76%)	337 (43.43%)
Disability	20 (0.51%)	11 (0.46%)	2 (0.26%)	–	7 (0.90%)
RI	2 (0.05%)	2 (0.08%)	–	–	–
Other serious	983 (24.90%)	592 (24.62%)	206 (27.21%)	44 (25.88%)	195 (25.13%)
Time-to-event (days)					
Median (IQR)	288.00 (76.00,784.75)	399.50 (107.00,1054.00)	152.00 (36.00,383.50)	204.00 (46.00,503.75)	326.50 (92.50,882.50)
Min–max	0–5,144	0–5,144	0–2,414	0–1,421	0–4,665

^§^Since there may be more than one outcome in a single report, the final level of seriousness for the single report is based on the following orders as recommended by FDA: death>life threatening>hospitalization>disability>required intervention to prevent permanent impairment/damage >other serious. FAERS, FDA Adverse Event Reporting System. IQR, interquartile range; RI, required intervention to prevent permanent impairment/damage.

### 3.3 Disproportionality analysis

The results of the overall disproportionality analyses are shown in **Table 2**. All the four biological classes had an elevated and similar reporting rates for MACEs relative to other alternative psoriasis treatments (PRR from 2.10 to 4.26 and EB05 from 1.15 to 2.45).

**Table 2:** Results of disproportionality analysis based on adverse event and MACEs case reporting to FAERS (2004 Q1–2022 Q4).

	Cases	PRR (χ2)	EBGM (95% two-sided CI)
Total			
MACE	4,206	2.41 (485.41)	1.24 (1.15,1.34)
Myocardial infarction	1,846	2.86 (296.27)	1.28 (1.13,1.44)
Stroke	1,188	2.31 (137.08)	1.23 (1.08,1.41)
Cardial fatality	1,399	2.20 (114.15)	1.22 (1.08,1.38)
Death	68	2.62 (26.18)	1.26 (0.70,2.28)
TNF inhibitors			
MACE	2,518	2.10 (272.60)	1.28 (1.19,1.39)
Myocardial infarction	1,146	2.58 (207.11)	1.35 (1.19,1.53)
Stroke	629	1.78 (46.13)	1.23 (1.06,1.42)
Cardial fatality	867	1.98 (66.71)	1.26 (1.11,1.44)
Death	48	2.69 (4.62)	1.36 (0.74,2.51)
IL-17 inhibitors			
MACE	851	2.57 (317.23)	1.79 (1.63,1.97)
Myocardial infarction	330	2.69 (140.37)	1.84 (1.58,2.14)
Stroke	279	2.86 (140.89)	1.89 (1.60,2.24)
Cardial fatality	320	2.65 (114.13)	1.82 (1.56,2.13)
Death	9	1.83 (0.17)	1.49 (0.64,3.48)
IL-23 inhibitors			
MACE	194	2.47 (97.24)	2.20 (1.88,2.58)
Myocardial infarction	86	2.96 (68.50)	2.55 (2.01,3.24)
Stroke	50	2.16 (19.55)	1.97 (1.46,2.68)
Cardial fatality	65	2.27 (22.06)	2.06 (1.57,2.69)
Death	3	2.57 (0.68)	2.27 (0.65,7.98)
IL-12/23 inhibitors			
MACE	808	4.26 (910.40)	2.69 (2.45,2.97)
Myocardial infarction	361	5.15 (518.28)	2.96 (2.55,3.44)
Stroke	260	4.66 (343.82)	2.82 (2.37,3.35)
Cardial fatality	211	3.06 (126.01)	2.24 (1.88,2.66)
Death	18	6.38 (23.27)	3.26 (1.60,6.65)

MACEs, major cardiovascular events; FAERS, US Food and Drug Administration Adverse Event Reporting System.

#### 3.3.1 Myocardial infarction

Myocardial infarction was the most significant signal generated. According to the criteria of the two data mining algorithms, all the four biological classes yielded positive and similar signals for myocardial infarction. IL-12/23 inhibitors demonstrated the highest PRR and EBGM (PRR, χ^2^518.28; EB05, 2.55), while TNF inhibitors showed the weakest association (PRR, 2.58 χ^2^207.11; EB05, 1.19).

#### 3.3.2 Stroke

Statistically, an increased reporting rate for stroke was observed for IL-12/23 inhibitors, IL-17 inhibitors, and IL-23 inhibitors in the descending order of association. TNF inhibitors indicated a non-significant trend toward higher-than-expected reporting rates, as they did not meet the PRR algorithm criteria, although they met the criteria of the MGPS algorithm (PRR 178, χ^2^46.13; EB05, 1.06).

#### 3.3.3 Cardiac fatality

The association pattern for cardiac fatality was similar with that for stroke (IL-12/23 inhibitors> IL-17 inhibitors> IL-23 inhibitors, without much difference). TNF inhibitors also revealed a non-significant trend toward higher-than-expected reporting rates.

#### 3.3.4 Death

Across the four biological classes, only IL-12/23 inhibitors were observed with a significantly increased reporting rate for mortality based on the PRR and EBGM criteria (PRR 638, χ^2^23.27; EB05, 1.60). TNF, IL-23, and IL-17 inhibitors did not meet the criteria of either the PRR or MGPS algorithms.

### 3.4 Drug–MACEs associations by age group

**Figure 1:**
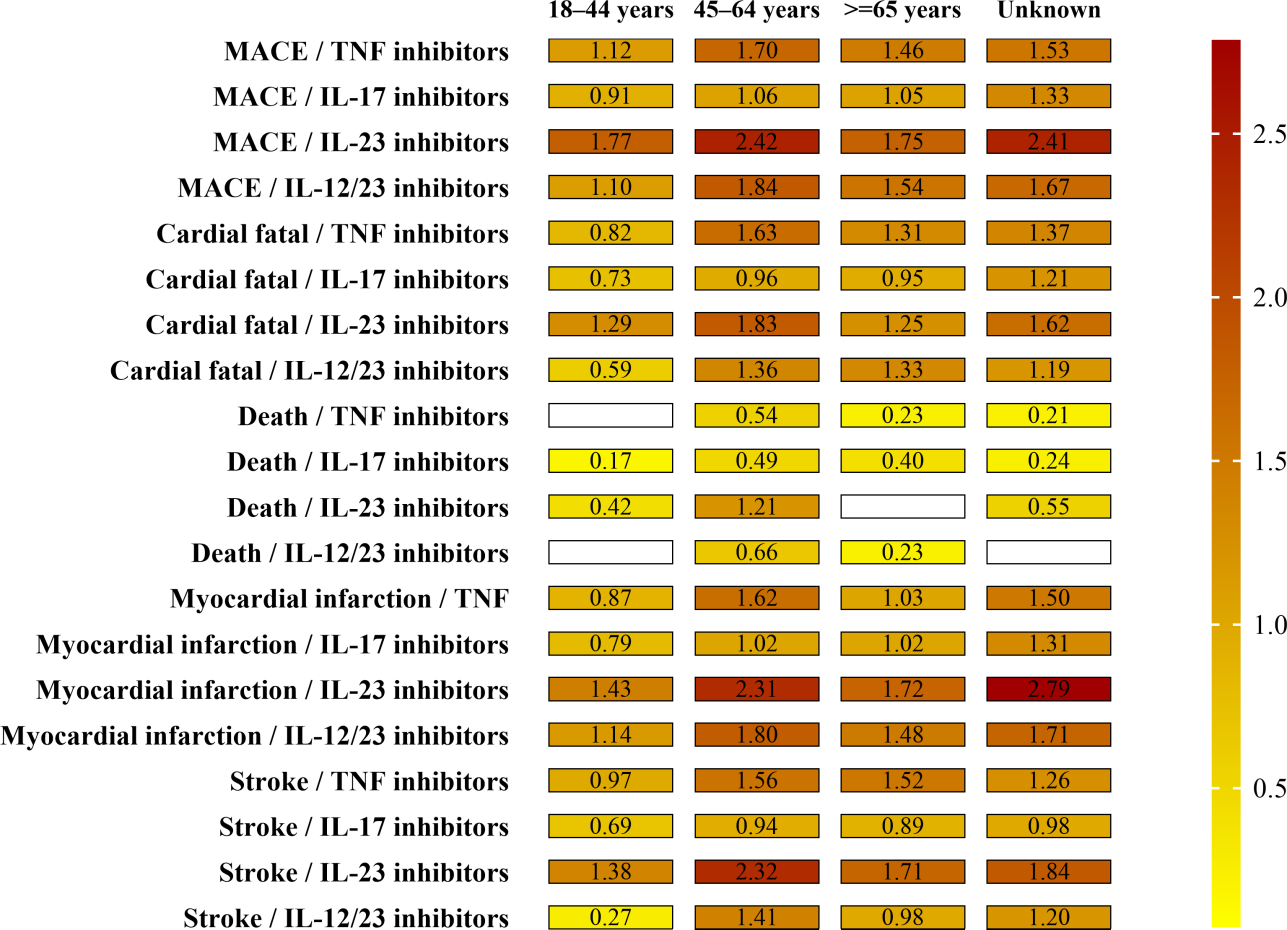
Paired EBGM values by drug and age groups for MACEs. Note the signal strength (EB05 value by color and the numerical EB05 value being within each minigraph). The higher the EBGM value, the higher probability of association between a drug and an adverse event.

The result of age cohort MGPS analysis is shown in **Figure 1** and **Supplementary Table S3**.

For children and adolescents (≤17 years), three cases of the four SMQs were identified for individuals, <17 years of age and receiving IL-17 inhibitors (1 case) and IL-12/23 inhibitors (2 cases). No signal of disproportionate reporting for MACEs was identified for any biologics.

For young-adults (18–44 years), a total of 377 cases of MACEs were found for individuals, aged 18–44 years, receiving four biological classes. Results for this cohort were similar to the total population; IL-17 inhibitors and IL-12/23 inhibitors demonstrated the highest frequencies of MACEs (238 and 82 cases, respectively) and only IL-12/23 inhibitors demonstrated an EB05 score>1 for the four SMQs (except death). Of the MACEs, myocardial infarction (161 cases) and cardiac fatality (118 cases) were the most frequently reported MACEs in this age group.

For middle adults (45–64 years), a total of 1,810 cases of MACEs were found for individuals, aged 45–64 years, receiving four biological classes. Results for this cohort were a little higher to the total population. Of all the four biological classes, only IL-12/23 inhibitors demonstrated an EB05 score>1 for the four SMQs (except death). Of the MACEs, myocardial infarction (864 cases) and cardiac fatality (540 cases) were the most frequently reported MACEs in this age group.

For the elderly (over 65 years), a total of 1,080 cases of MACEs were found for individuals, over 65 years of age, receiving four biological classes. Results for this cohort were similar to the total population. All the four biological classes demonstrated an EB05 score>1 for the certain SMQs, not for all the four SMQs. Of the MACEs, cardiac fatality (412 cases) and myocardial infarction (395 cases) were the most frequently reported MACEs in this age group.

### 3.5 Drug–MACEs associations by sex group

**Figure 2:**
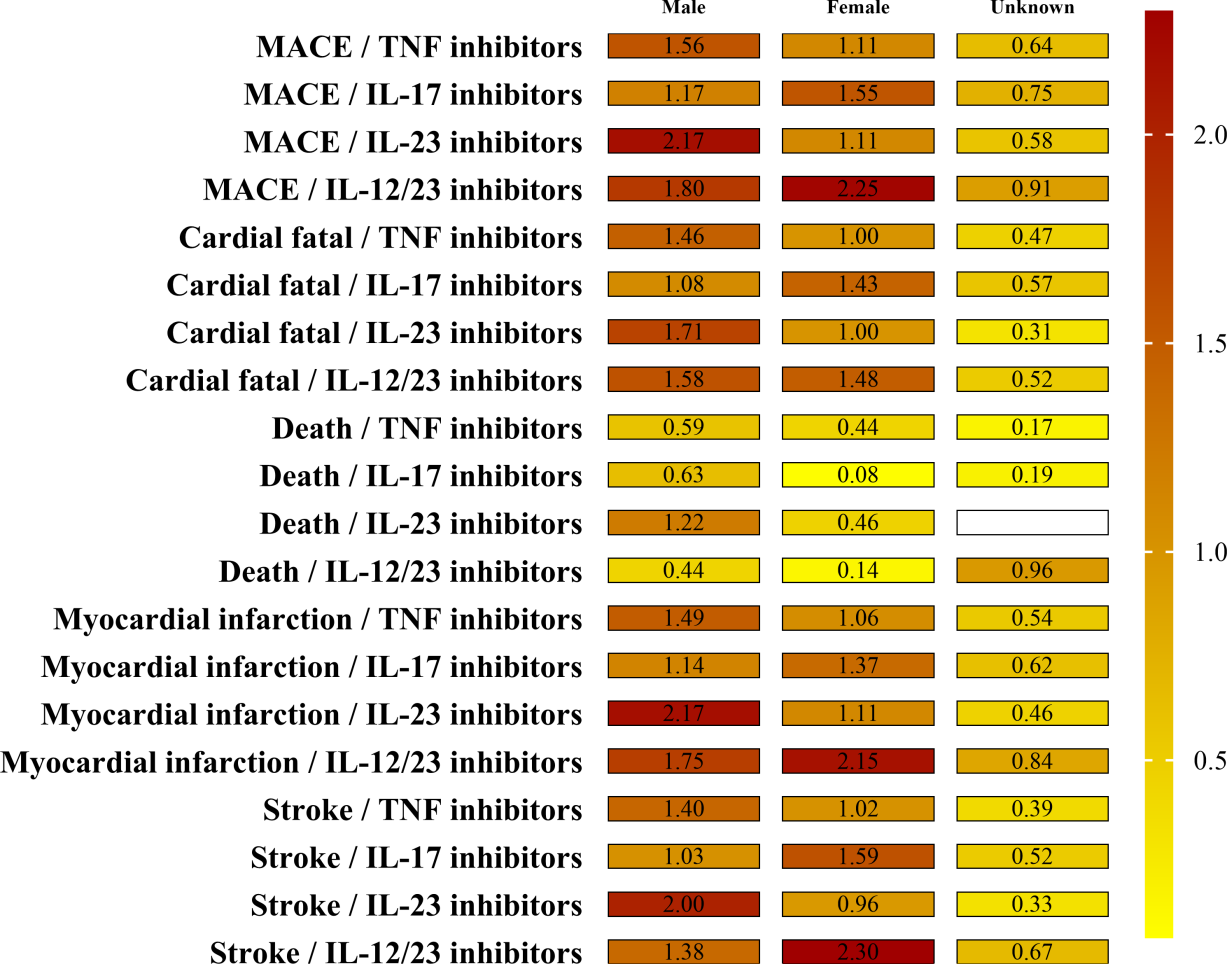
The EBGM values by sex groups and drug for MACEs. Note the signal strength (EB05 value by color and the numerical EB05 value being within each minigraph). The higher the EBGM value, the higher probability of association between a drug and an adverse event.

The result of sex cohort MGPS analysis is shown in **Figure 2** and **Supplementary Table S4**. Similar disproportionate MACEs reporting was seen between men and women although all the four biological classes demonstrated a little higher EB05 score>1 for the four SMQs in men than that in women. Among the four biological classes, IL-12/23 inhibitors had the highest EBGM values in males for all four SMQs: myocardial infarction (EB05 2.17), stroke (EB05 2.00), cardiac fatality (EB05 1.71), and death (EB05 1.22). As noted, most death cases associated with IL-12/23 inhibitors were reported in men (14 cases vs. 1 case).

### 3.6 Time to adverse drug reaction

**Figure 3:**
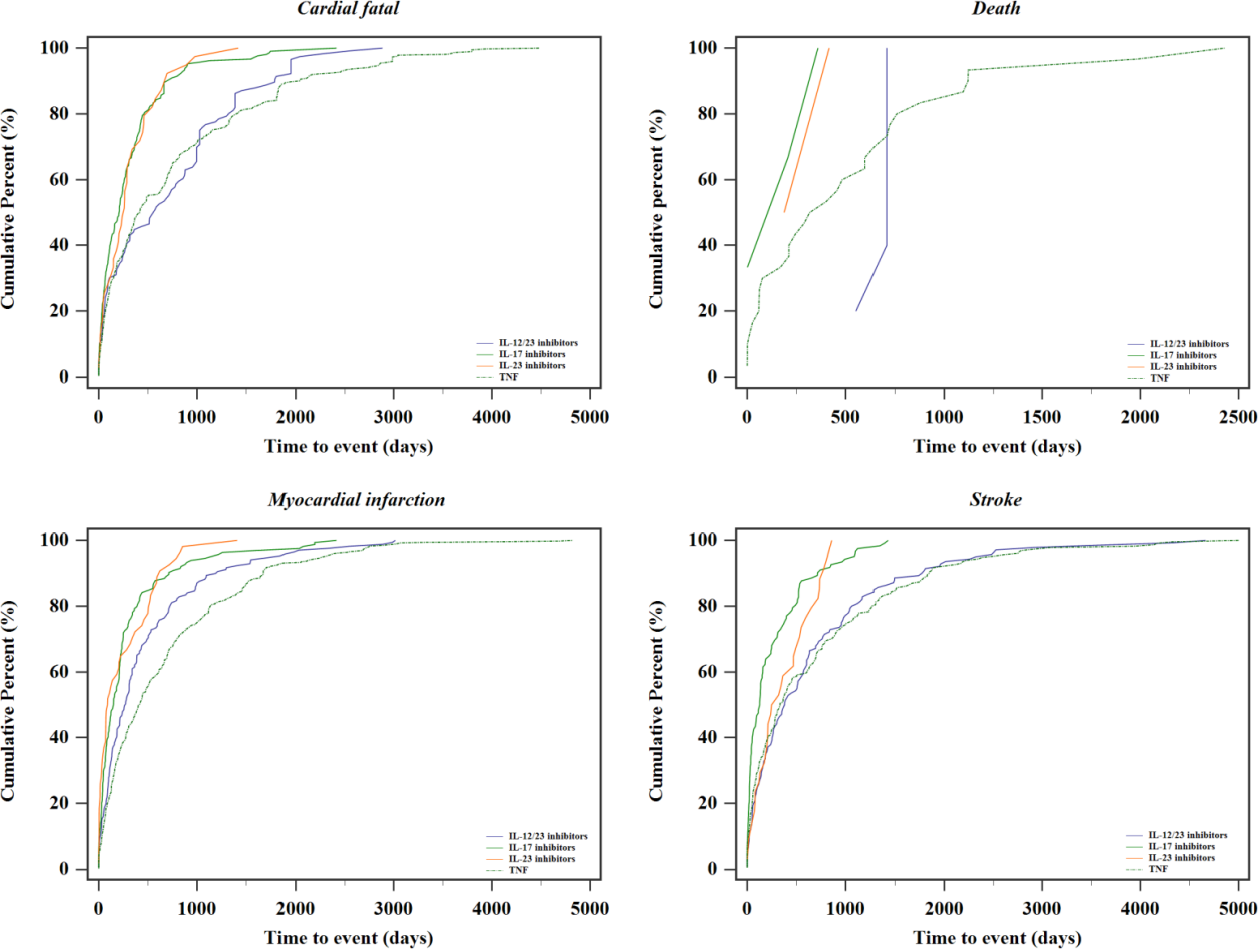
Cumulative frequency graph for the time to onset of MACEs associated with biologics.

The time to onset following each MACEs SMQ and individual biologic drug are summarized in **Figure 3**. Based on the available data of START_DT and EVENT_DT, the median time to the occurrence of the composite MACEs was 288 [IQR 76–784.75] days. Additionally, patients receiving TNF inhibitors had a numerically longer median time to onset of MACEs (399.50 days; IQR, 107.00–1,054.00 days), and 71.73% of MACEs events for this group occurred after 6 months.

## 4 Discussion

There is a growing concern that patients treated with biological agents may suffer an increased risk for MACEs. Pre-market clinical studies designed with the biological therapies produced favorable safety results. Given that most of the included studies were with short-term follow-up (≤12 months) and underpowered to detect rare safety events, and most study subjects were strictly selected to exclude older or those with more co-morbidities often seen in clinical practice, the conclusions from clinical trial safety data are limited. Accordingly, large-scale, real-world studies offer the ability to identify safety signals of infrequent adverse events and events that may manifest only after prolonged exposure to medication.

Utilizing real-world data from FAERS, this study revealed a significant association between the four biological psoriasis therapies and disproportionate reporting of MACEs when compared to all other systemic drugs. Myocardial infarction was the most significant signals generated. According to the results of PRR and MGPS algorithms, IL-12/23 inhibitors demonstrate the highest disproportionality ratio of all MACEs SMQs.

We noted patients with psoriasis have higher rates of cardiovascular diseases than the general population, regardless of treatment, due to the involvement of pro-inflammatory cytokines produced in psoriasis (35–37). This increased background risk of cardiovascular events in inflammatory conditions is postulated to increase the number of irrelevant cardiovascular event reporting with biologic agents, relative to other medical therapies for patients without such baseline risk factors. In an effort to mitigate the potential effect of underlying disease on MACEs outcomes, several measures were implemented including 1) restricting the study population (cases and controls) to patients with psoriasis. It can minimize the effect of confounding by indication and thereby create more balanced groups and reduce selection bias (38) and 2) using other systemic psoriasis medications except biologics as active comparators. Using active comparator medication with indication similar to the drugs under investigation has been shown to mitigate the influence of confounding factors related to the diseases through balancing the study groups and to improve the quality of comparison. This method has been implemented as an important analytical method in more recent post-marketing pharmacovigilance studies (28, 39, 40).

Theoretically, biological therapies treating psoriasis are presumed to ameliorate atherosclerosis and decrease the cardiovascular risk by blocking the inflammatory cytokines. However, other experimental data and pre-clinical trials do not support a protective effect of biologics against atherosclerotic plaque development (41, 42). Some researchers (2, 43) summarized the available data about the MACEs risk for the biological products and concluded that TNF inhibitors might reduce the risk of adverse cardiovascular events, the only IL-12/23 inhibitor ustekinumab appeared to be neutral, and the IL-17 inhibitors and IL-23 inhibitors were unclear, although more researches are needed to confirm these findings. A review of current data suggested positive or neutral effect of TNF, IL-17, and IL-12/23 inhibitors on cardiovascular events, but conflicting for IL-23 inhibitors (14). Contradictory findings emerge from real-world studies on the cardiovascular safety of biological therapies. Some studies reported no safety issues beyond those observed in pre-registration trials (44). In contrast, more recent observational pharmacovigilance studies highlighted an increased reporting of cardiovascular/cerebrovascular accidents associated with biological therapies (20, 45–48). The detailed information about these studies can be seen from **Table 3**. For example, recently, an analysis from FAERS database unveiled disproportionate reporting of cardiovascular adverse effects (including coronary artery disease, pericarditis, and atrial fibrillation) in association with novel psoriasis agents.

**Table 3:** Cardiovascular/cerebrovascular adverse events associated with biological therapies from pharmacovigilance studies.

No.	Title of the Study	Cardiovascular/ cerebrovascular events	Ref.
1	Increased reporting of cerebrovascular accidents with use of risankizumab observed in the Food and Drug Administration Adverse Events Reporting System (FAERS)	An increase in reporting of cerebrovascular accidents following use of risankizumab for psoriasis was revealed.	(48)
2	Potential cerebrovascular accident signal for risankizumab: A disproportionality analysis of the FDA Adverse Event Reporting System (FAERS)	A disproportionate reporting of myocardial infarction was associated with risankizumab compared with other drugs.	(20)
3	Adverse events with risankizumab in the real world: post-marketing pharmacovigilance assessment of the FDA adverse event reporting system	Unexpected cardiovascular adverse events such as myocardial infarction was found in association with risankizumab.	(45)
4	Pharmacovigilance of Risankizumab in the Treatment of Psoriasis and Arthritic Psoriasis: Real-World Data from EudraVigilance Database	Significant reporting of cardiac disorders were associated with risankizumab, and the most frequent adverse events was myocardial infarction.	(46)
5	Novel Psoriasis Agents Associated Cardiotoxicity: Analysis of FAERS	This study uncovers unknown cardiovascular adverse effects (including coronary artery disease and atrial fibrillation) related to biologic agents used in psoriasis treatment.	(47)

Under this active comparator-restricted approach, our data were in some way in agreement with some studies (6, 37, 38) in showing the highest disproportionate reporting of IL-12/23 inhibitors for MACEs, then IL-17 inhibitors and IL-23 inhibitors, and the lowest association for TNF inhibitors. In the EXCEED study (49), a head-to-head trial, comparing secukinumab (an IL-17 inhibitor) vs. adalimumab (a TNF-α inhibitor) for treatment of active psoriatic arthritis, reported two MACEs in the secukinumab group (n=426) and none in the adalimumab group(n=427). During UNCOVER-2 and UNCOVER-3 trials (50) where ixekizumab (an IL-17 inhibitor) was versus etanercept (a TNF-α inhibitor) or placebo with psoriasis, two MACEs events were reported in the ixekizumab group. In a recent nationwide cohort study (6), researchers reviewed the healthcare database of the French health insurance from 2015 to 2019, including 9,510 biologics users. They found that the risk of MACEs was significantly higher with IL-12/23 inhibitors, followed by IL-17 inhibitors when vs. TNF inhibitors. Given the different cardiovascular comorbidities or risk factors between patients, our study may provide help to weigh the efficacy and safety profile of various biological strategies when approaching real-life patients and their comorbidities (51).

The mechanism underlying this comparative result remains unclear (52). One possible hypothesis is that IL-17 plays a protective stabilizing role in atherosclerosis. Experimental evidence and biomarker studies (13, 53, 54) have indicated that instability in atherosclerotic plaque was linked to decreased levels of circulating IL-17, contributing to a higher risk of cardiovascular diseases. This is reinforced by a prospective trial, which enrolled 981 patients and highlighted that low serum levels of IL-17 were linked with a higher risk of all-cause death and recurrent myocardial infarction in Caucasian patients after 2-year follow-up (55). T helper (Th) 17 lymphocytes, a subfamily of CD4+ lymphocytes, produce IL-17, while IL-23 can stimulate IL-17 production by Th 17 lymphocytes (56). As we know, IL-12/23 inhibitors bind to both p19 and p40 subunits and hence dually block both IL-23 and IL-12 cytokines. On the other hand, selective IL-23 inhibitors bind to p19 subunit and only block IL-23 cytokine. Subsequently, they can directly decrease production of IL-17 and increase instability in atherosclerotic plaques. These findings underscore the importance of further research into the complex physiopathology of IL-17 and its implications in atherosclerosis, necessitating a greater focus on understanding these mechanisms.

In addition, our study reveals that ustekinumab, the only IL-12/23 inhibitor on the market, has the highest reporting rate for all four SMQs. This result is not surprising, and it raises concerns similar to the unlicensed IL-12/23 inhibitor, briakinumab, which discontinued its clinical trials before marketing due to high reports of MACEs (e.g., myocardial infarction) (18). It cannot be denied that the notoriety effect (57) after briakinumab withdrawal may exist and may serve to overestimate relevant MACEs events with IL-12/23 inhibitors in the treatment of plaque psoriasis. Considering the elevated reporting risk observed for myocardial infarction and cardiac fatality associated with IL-12/23 inhibitors in this study, additional analyses are warranted.

The MGPS analyses in this study suggest that different aged patients with psoriasis exhibit varying EBGM values for MACEs, with older patients (≥45 years) demonstrating a relatively higher risk of cardiovascular events. To note, the highest relative risk for psoriasis patients having an MACE occurs in 45–64 years subgroup, and the relative risk is attenuated in older. Despite the smaller relative number of MACEs reported in young adults (18–44 years), IL-12/23 inhibitors showed drug–event associations with three SMQs in this population (stroke, myocardial infarction, and cardiac fatality). Ultimately, physicians need to be aware of the risk of cardiac diseases in young adults receiving biological agents and take precautions, including detailed cardiovascular history and measures like electrocardiogram and cardiac ultrasonography, when they experience cardiovascular symptoms after the initiation of treatment (51). Confirmation of these preliminary observation analyses in further research is needed.

In addition, our study found that the risk for biologics-associated MACEs were similarly between men and women (EB05 1.12 and EB05 1.11, respectively). Differences in adverse events for psoriasis biologics between sexes have been studied in the past decades. A prospective study (58) did not identify significant differences in the occurrence of MACEs with biological treatment between female and male patients, although female patients reported more relevant adverse events with more fungal and herpes simplex infection. In contrast, some studies concluded that men had a higher incidence of MACEs compared to women (6, 59). Thus, deeper insight into gender differences in the specific types of adverse events during biological treatment is warranted.

Our study was the first to reveal time to event and discovered that the median time for the onset of drug–ADR pairs is 288 (IQR, 76–784.75) days, which much exceeds the duration observed in most current trials. Patients receiving TNF inhibitors and IL-12/23 inhibitors have a numerically higher median time to onset of events. It is similar to the previous investigations where the median time before MACEs was 12 (IQR, 5–22) months in studies involving biological disease-modifying antirheumatic drugs (DMARDs) (6). The current analysis did not show a clearly distinguishable “pattern” between the occurrence of MACEs and the treatment initiation. Although, in this analysis, the timing of ADRs seems widespread, this observed result must be interpreted with caution, since most therapy duration data were missing and the time to event can be potentially biased and requires further investigation.

The strengths of this post-marketing surveillance include the following: first, using the large-sample size database, FAERS, to investigate the disproportionate reporting of cardiovascular adverse events in association with biologic psoriasis agents; second, restricting the study population to patients with similar background risk factors and restricting the reference group to reports with active comparators. They can, in some degree, standardize baseline characteristics and further reduce channeling bias and enhance the efficacy of disproportionality. Third is conducting multiple subgroup MGPS analyses for all selected drug–event pairs based on age and sex to further reduce the influence of confounding variables (such as age and sex) on cardiovascular adverse events.

In the interpretation, the effect of some study limitations should be remembered. First, this study cannot calculate the incidence rate of adverse events because FAERS is a spontaneous adverse event reporting system that would capture voluntary post-marketing reports and cannot count the number of drug users (that is a denominator). Second, it is crucial to take into account the potential of a notoriety effect following the withdrawal of briakinumab, which may lead to an overestimation of relevant MACEs associated with IL-12/23 inhibitors when used for plaque psoriasis. Third, due to unavailable data of individual patient-level information including the cardiovascular history such as diabetes and nonfasting lipids, it is difficult to establish potential risk factors and to prevent the occurrence of MACEs; nonetheless, we try our best to made adjustments for age and sex.

## 5 Conclusions

Our study contributes to the existing literature by establishing the association between psoriasis and MACEs using a large FAERS dataset spanning from 2004 to 2022. Additionally, this study identifies impact of the age and sex on biologics-associated MACEs in an initial screening. This pharmacovigilance study suggests but not proves that individuals on biological agents tend to have an increased risk of MACEs in the psoriasis patients, and IL-12/23 inhibitors may be associated with the highest increased risk of MACEs. Patients aged 45–64 years are at higher risk of MACEs. Further comparative studies with larger sample size and longer follow-up duration are warranted to thoroughly explore the association between biologic psoriasis agents and MACEs and to identify any potential differences between the various biological classes.

## Data availability statement

The original contributions presented in the study are included in the article/**Supplementary Material**, further inquiries can be directed to the corresponding author/s.

## Author contributions

LD: Conceptualization, Data curation, Writing – original draft, Writing – review & editing. CC: Data curation, Formal analysis, Writing – review & editing. YY: Data curation, Methodology, Writing – review & editing. XZ: Investigation, Supervision, Validation, Writing – review & editing.
