# Supplementary material for: Major cardiovascular events under biologic psoriasis therapies: a 19-year real-world analysis of FAERS data
Source: Front Immunol. 2024 Feb 7;15:1349636. doi: 10.3389/fimmu.2024.1349636 (PMC10879569; doi:10.3389/fimmu.2024.1349636)
Supplement: Supplementary file 1 [file Table_1.docx]

Supplementary Material

# Table 1. Search terms for drug names of biologics treating psoriasis approved by FDA

| **Class** | **Generic name** | **Approved date** | **Brand name** |
| --- | --- | --- | --- |
| TNF-α inhibitors | Adalimumab | 12/31/2002 | Abrilada, Amjevita, Cyltezo, Hadlima, Hulio, Humira, Hyrimoz, Yusimry |
|  | Etanercept | 11/2/1998 | Enbrel, Erelzi, Eticovo |
|  | Infliximab | 8/24/1998 | Avsola, Inflectra, Ixifi, Remicade, Renflexis |
|  | Certolizumab | 4/22/2008 | Cimzia |
|  | Golimumab | 4/24/2009 | Simponi |
| IL-23 inhibitors | Guselkumab | 6/13/2017 | Tremfya |
|  | Risankizumab | 4/23/2019 | Skyrizi |
|  | Tildrakizumab | 3/20/2018 | Ilumya |
| IL-17 inhibitors | Ixekizumab | 3/22/2016 | Taltz |
|  | Secukinumab | 1/21/2015 | Cosentyx |
|  | Brodalumab | 2/15/2017 | Siliq |
| IL-12/23 inhibitors | Ustekinumab | 9/25/2009 | Stelara |

# Table 2. MACE categorization according to Medical Dictionary for Regulatory Activities (MedDRA) Classification Version 25.0

| **Group** | **MedDRA terms included** |
| --- | --- |
| Myocardial infarction | Myocardial infarction (SMQ broad 25.0) |
| Stroke | PT search from Central nervous system haemorrhages and cerebrovascular conditions (SMQ broad 25.0) |
| Cardiac fatal | Atrial fibrillation (PT 25.0) |
|  | Atrial flutter (PT 25.0) |
|  | Angina pectoris (PT 25.0) |
|  | Cardiac arrest (PT 25.0) |
|  | Cardiac failure (PT 25.0) |
|  | Cardio-respiratory arrest (PT 25.0) |
|  | Corpulmonale (PT 25.0) |
|  | Cardiopulmonary failure (PT 25.0) |
|  | Cardiogenic shock (PT 25.0) |
| Death | Sudden death (PT 25.0) |
|  | Cardiac death (PT 25.0) |
|  | Sudden cardiac death (PT 25.0) |

MACE:major adverse cardiovascular events. MedDRA, Medical Dictionary for Regulatory Activities. PT, preferred term. SMQ, Standardized MedDRA Query. SOC: system organ class.

# Table 3. Adjusted reporting ratios (Empiric Bayes Geometric Mean [EBGM] values) and 90% confidence intervals (EB05, EB95) for MACE associated with biologics for psoriasis by age groups

|  | 0-17 years | | 18-44 years | | 45-64years | | ≥65 years | | Unknown | |
| --- | --- | --- | --- | --- | --- | --- | --- | --- | --- | --- |
|  | No. of reports | EBGM  (EB05, EB95) | No. of reports | EBGM  (EB05, EB95) | No. of reports | EBGM  EB05, EB95) | No. of reports | EBGM  (EB05, EB95) | No. of reports | EBGM  (EB05, EB95) |
| **Total biologics** | | | | | | | | | | |
| MACE | 3 | - | 377 | 1.18(0.90,1.55) | 1810 | 1.20(1.07,1.35) | 1080 | 1.19(1.04,1.37) | 936 | 1.38(1.18,1.60) |
| Stroke | 2 | - | 101 | 1.19(0.70,2.02) | 511 | 1.22(0.97,1.53) | 343 | 1.20(0.94,1.52) | 231 | 1.29(0.99,1.68) |
| Myocardial infarction | 0 | - | 161 | 1.19(0.78,1.80) | 864 | 1.21(1.02,1.44) | 395 | 1.24(0.97,1.58) | 425 | 1.46(1.12,1.90) |
| Death | 0 | - | 13 | 1.28(0.17,9.78) | 29 | 1.18(0.49,2.85) | 14 | 1.09(0.39,3.03) | 12 | 1.55(0.20,11.95) |
| Cardiac fatality | 1 | - | 118 | 1.17(0.73,1.86) | 540 | 1.20(0.97,1.47) | 412 | 1.16(0.94,1.43) | 328 | 1.36(1.06,1.74) |
| **TNF inhibitors** | | | | | | | | | | |
| MACE | 0 | - | 54 | 1.61(1.12,2.33) | 351 | 1.56(2.77,1.97) | 200 | 1.76(1.46,2.13) | 246 | 1.85(1.53,2.22) |
| Stroke | 0 | - | 19 | 1.88(0.97,3.66) | 101 | 2.08(1.56,2.77) | 85 | 2.07(1.52,2.81) | 74 | 1.74(1.26,2.42) |
| Myocardial infarction | 0 | - | 21 | 1.55(0.87,2.75) | 166 | 2.77(1.97,1.70) | 45 | 1.49(1.03,2.15) | 98 | 2.05(1.50,2.81) |
| Death | 0 | - | 0 | - | 5 | 1.97(1.70,2.29) | 2 | 1.16(0.23,5.99) | 2 | 2.28(0.21,25.13) |
| Cardiac fatality | 0 | - | 17 | 1.55(0.82,2.94) | 125 | 1.70(2.29,2.02) | 85 | 1.74(1.31,2.30) | 93 | 1.86(1.37,2.51) |
| **IL-17 inhibitors** | | | | | | | | | | |
| MACE | 1 | - | 238 | 1.20(0.91,1.59) | 1076 | 1.20(1.06,1.35) | 674 | 1.22(1.05,1.41) | 529 | 1.56(1.33,1.83) |
| Stroke | 0 | - | 60 | 1.19(0.69,2.07) | 279 | 1.20(0.94,1.52) | 181 | 1.16(0.89,1.51) | 109 | 1.32(0.98,1.78) |
| Myocardial infarction | 0 | - | 106 | 1.22(0.79,1.87) | 523 | 1.22(1.02,1.46) | 275 | 1.32(1.02,1.69) | 242 | 1.72(1.31,2.26) |
| Death | 0 | - | 9 | 1.36(0.17,10.75) | 20 | 1.21(0.49,3.02) | 11 | 1.16(0.40,3.34) | 8 | 1.94(0.24,15.49) |
| Cardiac fatality | 1 | - | 75 | 1.18(0.73,1.92) | 326 | 1.19(0.96,1.49) | 264 | 1.18(0.95,1.47) | 201 | 1.57(1.21,2.04) |
| **IL-23 inhibitors** | | | | | | | | | | |
| MACE | 0 | - | 14 | 2.47(1.77,3.44) | 76 | 2.81(2.42,3.26) | 55 | 2.07(1.54,2.79) | 154 | 2.98(2.41,3.67) |
| Stroke | 0 | - | 2 | 2.60(1.38,4.91) | 19 | 3.06(2.32,4.04) | 14 | 1.74(0.98,3.07) | 44 | 2.68(1.84,3.91) |
| Myocardial infarction | 0 | - | 8 | 2.39(1.43,4.02) | 38 | 2.87(2.31,3.56) | 20 | 2.43(1.48,3.99) | 80 | 3.87(2.79,5.38) |
| Death | 0 | - | 0 | 3.62(0.42,30.97) | 2 | 3.34(1.21,9.19) | 1 | 1.98(0.23,16.99) | 3 | 5.26(0.55,50.60) |
| Cardiac fatality | 0 | - | 4 | 2.31(1.29,4.16) | 21 | 2.44(1.83,3.24) | 24 | 2.07(1.33,3.23) | 40 | 2.38(1.62,3.49) |
| **IL-12/23 inhibitors** | | | | | | | | | | |
| MACE | 2 | - | 82 | 1.97(1.10,3.54) | 379 | 2.37(1.84,3.05) | 190 | 2.12(1.75,2.57) | 49 | 2.28(1.67,3.12) |
| Stroke | 2 | - | 24 | 1.19(0.27,5.19) | 118 | 2.32(1.41,3.81) | 72 | 2.35(1.71,3.24) | 15 | 2.10(1.20,3.67) |
| Myocardial infarction | 0 | - | 32 | 2.52(1.14,5.58) | 178 | 2.58(1.80,3.69) | 70 | 2.38(1.72,3.30) | 20 | 2.83(1.71,4.68) |
| Death | 0 | - | 5 |  | 10 | 3.26(0.66,16.16) | 0 |  | 0 |  |
| Cardiac fatality | 0 | - | 24 | 1.72(0.59,5.01) | 88 | 2.17(1.36,3.48) | 59 | 1.72(1.25,2.36) | 16 | 2.05(1.19,3.51) |

MACE: major adverse cardiovascular events.

# Table 4. Adjusted reporting ratios (Empiric Bayes Geometric Mean [EBGM] values) and 90% confidence intervals (EB05, EB95) for MACE associated with biologics for psoriasis by gender

|  | **Male** | | **Female** | | **Unknown** | |
| --- | --- | --- | --- | --- | --- | --- |
|  | **No. of reports** | **EBGM** | **No. of reports** | **EBGM** | **No. of reports** | **EBGM** |
|  |  | **(EB05, EB95)** |  | **(EB05, EB95)** |  | **(EB05, EB95)** |
| **Total biologics** | | | | |  |  |
| MACE | 2565 | 1.24(1.12,1.37) | 1478 | 1.24(1.11,1.39) | 163 | 1.01(0.68,1.52) |
| Stroke | 649 | 1.23(1.01,1.49) | 497 | 1.24(1.02,1.51) | 42 | 1.00(0.47,2.13) |
| Myocardial infarction | 1198 | 1.26(1.08,1.47) | 559 | 1.29(1.06,1.57) | 89 | 1.04(0.58,1.86) |
| Death | 47 | 1.25(0.59,2.64) | 16 | 1.19(0.44,3.25) | 5 | - |
| Cardiac fatality | 851 | 1.22(1.03,1.45) | 499 | 1.21(1.00,1.45) | 49 | 1.00(0.49,2.04) |
| **TNF inhibitors** | | | | |  |  |
| MACE | 1534 | 1.30(1.17,1.44) | 891 | 1.26(1.11,1.42) | 93 | 1.21(0.75,1.96) |
| Stroke | 357 | 1.26(1.03,1.55) | 253 | 1.19(0.96,1.48) | 19 | 1.25(0.52,3.01) |
| Myocardial infarction | 726 | 1.34(1.14,1.57) | 371 | 1.36(1.11,1.67) | 49 | 1.24(0.62,2.49) |
| Death | 33 | 1.36(0.63,2.95) | 13 | 1.30(0.46,3.64) | 2 | - |
| Cardiac fatality | 530 | 1.29(1.08,1.54) | 306 | 1.22(1.00,1.48) | 31 | 1.29(0.57,2.92) |
| **IL-17 inhibitors** | | | | |  |  |
| MACE | 480 | 1.77(1.56,2.02) | 329 | 1.80(1.55,2.09) | 42 | 1.80(1.55,2.09) |
| Stroke | 128 | 1.79(1.40,2.30) | 138 | 2.03(1.59,2.58) | 13 | 2.03(1.59,2.58) |
| Myocardial infarction | 208 | 1.82(1.49,2.21) | 101 | 1.79(1.37,2.34) | 21 | 1.79(1.37,2.34) |
| Death | 7 | 1.63(0.59,4.48) | 1 | 0.65(0.08,5.57) | 1 | 0.65(0.08,5.57) |
| Cardiac fatality | 177 | 1.81(1.46,2.25) | 127 | 1.82(1.43,2.31) | 16 | 1.82(1.43,2.31) |
| **IL-23 inhibitors** | | | | | | |
| MACE | 124 | 2.20(1.80,2.70) | 56 | 1.99(1.50,2.64) | 14 | 1.50(0.91,2.46) |
| Stroke | 30 | 2.07(1.38,3.09) | 16 | 1.74(1.03,2.92) | 4 | 1.61(0.67,3.90) |
| Myocardial infarction | 56 | 2.36(1.75,3.19) | 23 | 2.53(1.62,3.96) | 7 | 1.66(0.84,3.30) |
| Death | 2 | 2.08(0.44,9.81) | 1 | 2.60(0.30,22.29) | - | - |
| Cardiac fatality | 44 | 2.21(1.58,3.09) | 17 | 1.63(0.98,2.69) | 4 | 1.30(0.52,3.29) |
| **IL-12/23 inhibitors** | | | | | | |
| MACE | 538 | 2.46(2.17,2.79) | 232 | 2.65(2.25,3.13) | 38 | 1.11(0.58,2.11) |
| Stroke | 152 | 2.55(2.00,3.24) | 95 | 3.00(2.30,3.92) | 13 | 1.11(0.33,3.68) |
| Myocardial infarction | 257 | 2.62(2.17,3.17) | 81 | 2.85(2.15,3.79) | 23 | 1.16(0.46,2.92) |
| Death | 14 | 2.91(1.22,6.94) | 1 | 1.17(0.14,10.05) | 3 | - |
| Cardiac fatality | 144 | 2.14(1.71,2.69) | 58 | 2.00(1.48,2.72) | 9 | 1.02(0.31,3.32) |

MACE:major adverse cardiovascular events.
